# Functional Outcome Measurement in Patients with Lower-Extremity Soft Tissue Sarcoma: A Systematic Literature Review

**DOI:** 10.1245/s10434-019-07698-w

**Published:** 2019-08-12

**Authors:** Gilber Kask, Ian Barner-Rasmussen, Jussi Petteri Repo, Magnus Kjäldman, Kaarel Kilk, Carl Blomqvist, Erkki Juhani Tukiainen

**Affiliations:** 1 Department of Plastic Surgery, University of Helsinki and Helsinki University Hospital, HUS, Helsinki, Finland; 2 Department of Orthopaedics and Traumatology, Unit of Musculoskeletal Surgery, Tampere University Hospital, Tampere, Finland; 3 Department of Surgery, Central Finland Central Hospital, Jyväskylä, Finland; 4 Helsinki University Hospital Comprehensive Cancer Center, HUS, Helsinki, Finland

## Abstract

**Background:**

The importance of functional outcome (FO) in the treatment of patients with extremity soft tissue sarcoma (STS) has been increasingly recognized in the last three decades. This systematic review aimed to investigate how FO is measured in surgically treated lower-extremity STS patients.

**Methods:**

A systematic search of PubMed, Web of Science, and Scopus was performed based on the PRISMA guidelines. The methodologic quality of the publications was measured using the MINORS tool. The results from the included studies examining measurement types, measures, and time of FO measurement were compiled. The FO pooled mean and standard deviation were calculated as a weighted average for the groups. The validity of the applied measures is reported.

**Results:**

The literature search found 3461 publications, 37 of which met the inclusion criteria. The measurement types used were clinician-reported outcomes (*n* = 27), patient-reported outcomes (*n* = 20), and observer-reported outcomes (*n* = 2). The most frequently used measures were the Toronto Extremity Salvage Score (TESS) (*n* = 16) and the Musculoskeletal Tumor Society (MSTS) score 1993 (*n* = 12). The postoperative FO was relatively good. The pooled mean TESS and MSTS 1993 scores were respectively 83.3 and 86.2 (out of 100). Of the 10 previously reported measures, 3 provide validated FO scores. The methodologic quality of publications was generally low.

**Conclusions:**

Based on this systematic review, several different methods exist for assessing FO in patients with lower-extremity sarcoma. The most frequently used measure is a validated TESS. The postoperative FO of patients with lower-extremity STS seems to increase to the preoperative baseline level during long-term follow-up evaluation.

In the last 30 years, limb salvage has become the standard of care in the treatment of extremity sarcoma, and amputations are rare. This has been achieved by improved diagnostics, pre- and postoperative radiotherapy, and more refined reconstructive surgical methods. Reports on functional outcome (FO) have been increasing.^1^–^3^

Several methods for assessing FO have been described, including subjective and objective measures.^2^–^4^ Functional outcome measures should be valid, reliable, accurate, and clinically meaningful for the population in which the measurement is made.^5^,^6^ Consistent usage of the same measures allows benchmarking and comparison of study results over time and between research centers.

A few previous review studies have considered the topic from different perspectives. The review by Davis^4^ focused on FO of all extremity tissue sarcoma patients, including upper extremities and bone sarcomas. Tang et al.^3^ investigated quality-of-life studies in adult extremity sarcomas (also including upper extremities and bone sarcomas) and all quality-of-life studies. Furtado et al.^2^ reviewed physical functioning after treatment for lower- and upper-extremity sarcoma patients, including both bone and soft tissue sarcoma (STS). Only objective measures investigating postural balance, gait, and physical activity were included. All patient-reported outcome (PRO) measurement studies were excluded. Groundland et al.^7^ investigated pediatric patients. Wilson et al.^8^ studied pelvic sarcoma patients, and Winnette et al.^9^ investigated all patients with STS, including abdominal sarcomas. Thus, no systematic literature review has previously focused specifically on measurement of FO after surgical treatment of adult lower-extremity STS patients.

This study aimed to identify how FO has been measured in patients with surgically treated lower-extremity STS. More specifically, we sought to determine the type of methods and measures used to measure FO, whether the measures used had been tested for validity, FO for lower-extremity STS patients, and quality of the publications that report FO.

## Materials and Methods

### Overview and Eligibility Criteria for Review

A systematic literature review was performed based on the Preferred Reporting Items for Systematic Reviews and Meta-Analyses (PRISMA) guidelines.^10^ A review protocol was created by the authors and is available on request.

The study included all publications concerning patients with surgically treated lower-extremity STS whose FO was measured. The exclusion criteria ruled out duplication, studies that included fewer than 20 lower-extremity STS patients (considered pilot studies),^11^ non-adult study populations, and publications in languages other than English.

In the current review, the measures for assessing FO were classified as “previously developed and reported measures” or “new measures developed by the authors.” The “previously developed and reported measures” were defined as measures developed to assess FO and published previously. Measures, scales, or questionnaires developed by the authors themselves with the purpose to assess FO only in the reviewed article were considered “new measures developed by the authors.”

To report the type of measurement applied for assessment of FO, we used the terms “patient-reported outcome,” “clinician-reported outcome,” “observer-reported outcome,” and “performance outcome” measures.^12^ Patient-reported outcome (PRO) measures are based on a patient’s subjective assessment. Clinician-reported outcome measures are based on evaluation by a trained health care professional. Observer-reported outcome measures are based on observation by a person other than the patient or a health professional. Performance outcome is based on measurement of performance in specific tasks and are considered objective measures.

### Search Methods

PubMed, Scopus, and Web of Science search engines were used for the search. All published articles were retrieved without a search time constraint on 5 May 2018. The keywords combined with Medical Subject Headings (MeSH) terms were “lower AND (limb OR limbs OR leg OR legs OR extremity OR extremities OR foot) AND sarcoma AND (functional OR functionality OR function OR outcome).”

Two authors (G.K. and M.K.) independently reviewed all titles and appropriate abstracts. All unsuitable articles were excluded by the previously mentioned exclusion criteria. A manual search was performed for all references of suitable studies by review of titles and appropriate abstracts. The included studies were reviewed and added to the final list by the set inclusion criteria. Disagreements in data extraction were resolved by discussion and consensus of the authors (G.K., M.K., K.K., J.R., and I.B.R.).

### Study Data

Two authors (G.K. and M.K.) independently collected the following information from the included publications: study period, origin of the study, article type, anatomic location of the tumor, number of patients, age, diagnosis, measures and measurement types used for assessing FO, results of FO, and follow-up time. In case of missing data, an e-mail requesting additional information was sent to the corresponding authors.

### Validity Assessment

In this review, a validated FO measure is defined as a measure that has been scientifically validated to assess FO in extremity tumor patients. A literature search was performed to detect suitable literature concerning the validity of the FO measures.

### Functional Outcome

In this review, FO is reported from publications that used validated FO measures, and FO results are presented as means ± standard deviations (SDs). The pooled mean and SD were calculated as a weighted average of SDs for the groups. Publications reporting FO for bone sarcoma or upper-extremity STS patients in addition to lower-extremity STS patients were included in the FO report.

### Quality of Publications Assessment

Because the quality of a publication can be measured according to many different criteria, and because the validity of these criteria have not been determined, quality was not used to exclude studies in this review.^13^ The Methodological Index for Nonrandomized Studies (MINORS) quality assessment tool was used to assess the quality of publications.^14^ The MINORS tool is a valid instrument designed to assess the methodologic quality of nonrandomized surgical studies, either comparative or noncomparative.^14^ We used the MINORS tool to assess the quality of randomized controlled trials (RCTs), as has been done in previous literature.^15^

The MINORS tool consists of the following 12 methodologic items for studies: a clearly stated aim, inclusion of consecutive patients, prospective collection of data, end points appropriate for the aim of the study, unbiased assessment of the study end point, follow-up period appropriate for the aim of the study, less than a 5% loss to follow-up evaluation, and prospective calculation of the study size. Additional criteria for comparative studies include adequate statistical analyses, an adequate control group, contemporary groups, and baseline equivalence of groups. The items are scored as follows; 0 (not reported), 1 (reported but inadequate), or 2 (reported and adequate). The maximum score is 16 for non-comparative studies and 24 for comparative studies.^14^ Results are reported as percentages from 0 to 100.

## Results

### Study Selection

**Fig. 1:**
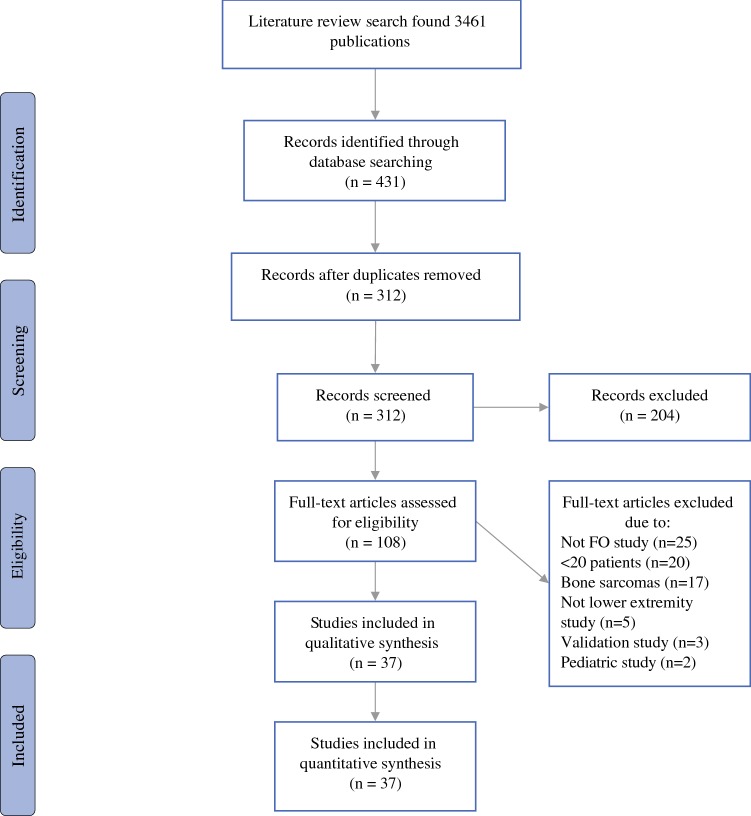
Flow diagram showing flow of studies retrieved for systematic review of functional outcome measurement in patients with lower-extremity soft tissue sarcoma

Details of the literature search are presented in Fig. 1. For the final review, 37 publications were selected.^16^–^52^

### Study Characteristics

The included studies were published between 1984 and 2018. Of the 31 retrospective and 6 prospective studies, 3 were RCTs. Of the 37 studies, 25 were cross-sectional, 7 were cohort, and 2 were case–control studies. The sample sizes of the studies ranged from 25 to 728 patients. The characteristics of the articles and the FO measurements are presented in Table 1.

**Table 1:** Characteristic of the articles and functional outcome measurements

Author	Year	Country	Study type	Patients	Tumor location	Number of patients	Age (years)^a^	Measure	Measurement type	Measurement time points	MINORS score
Slump et al.^18^	2018	NET/CAN	Re, C	STS, free or pedicled flap reconstruction	LE; UE	266. LE 181	59.2 ± 18.6	TESS, MSTS 1987, 1993	PRO, ClinRO	Pre- and postoperatively at 9–12 months	75
Podleska et al.^16^	2017	GER	Re, CS	STS, previously nonresectable sarcoma, chemotherapy	LE	27	52.7 (12–73)	SMFA-D	PRO	Minimum 5 months after surgery	69
Stoeckle et al.^17^	2017	F	Re, CS	STS, primary operated non-metastatic STS	LE	728, LE 480	Median 59 (16–91)	Own	ClinRO	N/P	44
Saebye et al.^19^	2017	DEN	Re, CS	STS, treated with first-time limb-sparing surgery without bone resection	LE; UE	128, LE 90	Median 61 (IQR 47–70)	TESS, MSTS 1993	PRO, ClinRO	Minimum 1 year after surgery	75
Harati^20^	2016	GER	Re; CS	STS, extending distally to the level of the knee joints	LE	120	57.4 (17–89)	TESS, FF I, SF–36	PRO	Minimum 1.3 years after surgery	50
Heaver et al.^21^	2016	UK	Re, CS	BTS/STS/other tumors, patients underwent limb-salvage surgery	LE; UE	198, LE 152	LE: 52.5 (14–86)	TESS	PRO	Minimum 3 months after surgery	38
Chang et al.^22^	2016	USA	Re, CS	All tumor types, 77 muscle only flaps, 67 MC flaps and 76 FC flaps	LE;UE	220. LE 156	LE: 49.5 ± 18.1	MSTS 1993, Karnof-sky score	ClinRO	N/P	71
Furtado et al.^23^	2015	UK	Re, CS	BTS/STS, primary tumor, management by primary or secondary amputation	LE	100, LE STS 37	53.6 (19–91)	TESS	PRO	Minimum 1 year after surgery	56
Rivard et al.^24^	2015	CAN	Pr, C	STS, primary or recurrent sarcoma	LE;UE; T	52, LE 35	57 (24–83)	TESS, MSTS 1993, SF–36	PRO, ClinRO	Preoperatively and at 6 and 12 months postoperatively	92
Kang et al.^25^	2014	S-K	Re, CC	STS, flap reconstruction case-matched with primary wound-closure group	LE; UE	148, LE 104	48.1 ± 19.3	MSTS 1993	ClinRO	12–15 Months after surgery	71
Townley et al.^26^	2013	CAN	Re, C	BTS/STS, preoperatively irradiated compared with non-irradiated patients		46, LE 24	57 ± 18	TESS, MSTS 1987	PRO, ClinRO	Minimum 1 year after surgery	63
Kozawa & Nishida^27^	2012	JAP	Re, CS	BTS/STS, foot sarcoma. Limb salvage surgery or amputation	LE	31, 24 STS	43 (11–76)	ISOLS	ClinRO	Minimum 2 years after surgery	56
Friedmann et al.^28^	2011	CAN	Re, C	STS, limb salvage surgery with or without adjuvant external beam radiotherapy	LE; UE	288. LE 207	53 (16–88)	MSTS 1993, TESS	PRO, ClinRO	Minimum 1 year after surgery	63
Thacker et al.^29^	2008	USA	Re, CS	STS, foot- and ankle-treated sarcoma patients	LE	52	38 (11–96)	MSTS 1993	ClinRO	Minimum 2 years after surgery	63
Tsukushi et al.^30^	2008	JAP	Re, CS	STS, investigation of results of arterial vs arteriovenous reconstruction	LE	25	46 (18–77)	MSTS 1993	ClinRO	Minimum 8 months after surgery	71
Hoy et al.^31^	2006	USA	Re, CS	BTS/STS/other, sarcoma resection, limb salvage and MFR		102 MFR, STS 36	39 (4–90)	MSTS 1993	ClinRO	Minimum 4 months after surgery	50
Pardasaney et al.^32^	2006	USA	Re, CS	BTS/STS, amputations and limb salvage	LE	408	49 (11–96)	Own	PRO, ObsRO	Minimum 2 years after surgery	71
Pradhan et al.^33^	2006	USA, CAN, UK	Re, CS	STS, treatment of the adductor compartment tumors, 3-center comparative study	LE	184	56 (13–88)	TESS	PRO	N/P	50
Nelson et al.^34^	2006	USA	Re, CS	STS, wide resection, functional restoration surgery or soft-tissue coverage with flaps	LE; UE	67, 48 LE	52 (13–84)	TESS	PRO	N/P	54
Thijssens et al.^35^	2006	NET	Re, CS	STS, limb salvage vs amputation	LE;UE	O 39, LE 33	Median 59 (15–78)	SF-36^b^	PRO	Minimum 1 year after surgery	75
Davis et al.^36^	2005	CAN	RCT	STS, surgery and pre- or postoperative radiotherapy. Function and radiation morbidity	LE; UE	129, LE 100	N/P	MSTS 1987, TESS	PRO, ClinRO	2 Years	88
Gerrand et al.^37^	2004	CAN	Re, C	STS, limb-sparing surgery	LE	207	54 (15–89)	MSTS 1993, TESS	PRO, ClinRO	Pre- and postoperatively within 1–2 years	71
Rachbauer et al.^38^	2003	AUS	Pr, CS	STS, marginal surgical resection, combined with both IOHDR and EBRT	LE; UP; T; R; HN	39, LE 29	Median 58 (17–87)	MSTS 1993	ClinRO	Minimum 3 months after surgery	69
O’Sullivan et al.^52^	2002	CAN	RCT	STS, randomized by tumor size dichotomized at 10 cm. Pre- and postoperative radiotherapy groups. Wound healing	LE; UE	182, LE 145	54.7	MSTS 1987, TESS, SF-36	PRO, ClinRO	Preoperatively and at 6 weeks and at 3, 6, 12 and 24 months postoperatively	96
Davis et al.^39^	2002	CAN	RCT	STS, randomized by tumor size dichotomized at 10 cm. Pre- and postoperative radiotherapy groups. Functional outcome	LE; UE	185, LE 147	54.7	MSTS 1987, TESS, SF-36	PRO, ClinRO	Preoperatively and at 6 weeks and 3, 6, 12 and 24 months postoperatively	96
Refaat et al.^40^	2002	USA	Re, CS	BTS/STS, limb salvage or amputation	LE	408	55	Own	PRO, ObsRO	N/P	67
Davis et al.^41^	2000	CAN	Re, CS	STS, limb-preservation surgery		172	51	MSTS 1987, 1993, TESS, SF-36	PRO, ClinRO	Minimum 1 year after surgery	56
Davis et al.^42^	1999	CAN	Re, CC	STS/BTS, amputation matched with limb-sparing surgery	LE	36	32	TESS, SF-36, RNL	PRO	Minimum 1 year after surgery	75
Colterjohn et al.^43^	1997	CAN	Re, C	STS/fibromatosis, limb-salvage surgery for foot and ankle	LE	30	51	MSTS 1987	ClinRO	See comment	56
Pitcher & Thomas^44^	1994	UK	Re, CS	STS, “functional compartmental resection”	LE	24	Median 47 (12–79)	Own	ClinRO	Minimum 1 month after surgery	50
Keus et al.^45^	1994	NET	Re, CS	STS, local treatment with curative intent and no known distant metastases	LE; UE	156, LE 115	Median 46 (4–89)	Own	ClinRO	N/P	38
Moseley^46^	1992	USA	Pr, C	STS, limb salvage using neoadjuvant chemotherapy, 2 groups	LE; UE	38, LE 27	Median 46/54^c^	Own	ClinRO	N/P	79
Karasek et al.^47^	1992	USA	Re, CS	STS/fibromatosis	LE; UE; T	41, LE 25	50 (13–85)	Own	ClinRO	Minimum 7 months after surgery	63
Robinson et al.^48^	1991	UK	Re, CS	STS, treated with combination of surgery and radiotherapy; surgery alone; irradiation and intra-arterial doxorubicin	LE; UE; P	54, LE 46	52 (23–82)	Own	ClinRO	Minimum 24 months after surgery	31
Stinson et al.^49^	1991	USA	Re, CS	STS, limb-sparing surgery, radiation therapy with or without adjuvant chemotherapy	LE; UE	145	N/P	Own	ClinRO	Minimum 1 year after surgery	38
Talbert et al.^50^	1990	USA	Re, CS	STS/unspecified, non-metastatic patients	LE; UE	78, LE 39	Age zones presented	Own	ClinRO	Minimum 28 months after surgery	44
Lampert et al.^51^	1984	USA	Re, CS	STS, wide local resection and radiation therapy	LE; UP; T; HN	40, 20 LE STS	37 (15–67)	Own, Convery scale	ClinRO	Minimum 7 months after surgery	56

*NET* The Netherlands, *CAN* Canada, *GER* Germany, *F* France, *DEN* Denmark, *UK* United Kingdom, *USA* United States of America, *S-K* South Korea, *JAP* Japan, *AUS* Australia, *Re* retrospective study, *C* cohort study, *CS* cross-sectional study, *Pr* prospective study, *CC* case–control study, *RCT* randomized control trial, *STS* soft tissue sarcoma, *BTS* bone tissue sarcoma, *IOHDR* intraoperative brachytherapy, *EBRT* external beam irradiation, *LE* lower-extremity, *UE* upper extremity, *T* trunk, *R* retroperitoneum, *HN* head and neck, *P* pelvic, *MFR* muscle flap reconstructions, *TESS* Toronto extremity salvage score, *MSTS* Musculoskeletal Tumor Society, *SMFA-D* the German Short Musculoskeletal Function Assessment questionnaire, *FFI* Foot Function Index, *ISOLS* International Society Of Limb Salvage, *RNL* Reintegration to Normal Living, *PRO* patient-reported outcome, *ClinRO* clinician-reported outcome, *ObsRO* observer-reported outcome

^a^Presented as mean age, unless otherwise stated

^b^Measure used was RAND-36

^c^Group A median, 46 years; Group B median, 54 years

### Type of Measurement to Assess FO

A single PRO measurement was used in 8 of the 37 publications, and a clinician-reported outcome measurement was used in 17 of the 37 publications. The PRO and clinician-reported outcome measurement types were used together in 10 studies. The PRO and observer-reported outcome measures were used in two studies. No performance outcome measurement was used. All six prospective studies used clinician-reported outcome measures, and four of these also used a PRO measure (Table 2).

**Table 2:** Previously developed and reported patient-reported outcome (PRO) and clinician-reported outcome (ClinRO) tools

Measure	Measurement type	*n*	Studies						Valid
			R (*n* = 31)	P (*n* = 6)	CS (*n* = 25)	C (*n* = 7)	CC (*n* = 2)	RCT (*n* = 3)	
TESS	PRO	16	12	4	7	5	1	3	+
MSTS 1993	ClinRO	12	10	2	7	4	1	0	+
MSTS 1987	ClinRO	7	4	3	1	3	0	3	+
SF-36	PRO	7	5	2	3	1	1	2	
SMFA	PRO	1	1	0	1	0	0	0	
FFI	PRO	1	1	0	1	0	0	0	
Karnofsky score	ClinRO	1	1	0	1	0	0	0	
Modified MSTS 1993 (ISOLS)	ClinRO	1	1	0	1	0	0	0	
RNL	PRO	1	1	0	0	0	1	0	
Convery scale	ClinRO	1	1	0	1	0	0	0	

*R* retrospective study, *P* prospective study, *CS* cross-sectional study, *C* Cohort study, *CC* Case–control study, *RCT* randomized clinical trial, *TESS* Toronto extremity salvage score, *MSTS* Musculoskeletal Tumor Society, *SMFA* Short Musculoskeletal Function Assessment, *FFI* Foot Function Index, *ISOLS* International Society Of Limb Salvage, *RNL* Reintegration to Normal Living, *PRO* patient-reported outcome, *ClinRO* clinician-reported outcome

### FO Measures

The majority of the publications (27/37) used previously reported measures. The most common measures were the Toronto Extremity Salvage Score (TESS) (43%, *n* = 16) and the Musculoskeletal Tumor Society (MSTS) score 1993 (32%, *n* = 12). A total of 10 different previously reported measurement tools were used (Table 2). New measures developed by the authors were used in 10 publications. Of the 27 publications, one previously reported measure alone was used in 14 publications, two and three measures in 6 publications each, and four measures in 1 publication.

The RAND-36 is multidimensional PRO questionnaire identical to the Short Form 36 (SF-36) but uses a different scoring method.^35^ In this review, RAND-36 and SF-36 are reported together.

### Timing of FO Measurement

In 4 (11%) of the 37 studies, FO was measured both pre- and postoperatively.^24^,^37^,^39^,^52^ More than one postoperative time point of measurement was used in 3 (8%) of the 37 studies.^24^,^39^,^52^ The time of FO measurement varied in the 31 retrospective studies. The inclusion criteria required a minimum follow-up period of 1 year in most of the studies. In 18 (58%) of the 31 retrospective studies and 12 (50%) of the 24 retrospective cross-sectional studies, the FO was measured at least 1 year after the surgery. The time point of measurement was not reported in seven studies, and additional information was not available. Additional details are presented in Table 1.

### Validity of the Measures

Validated FO measures were used in 23 (62%) of the 37 publications. the validated measures were the TESS^1^,^53^ and MSTS 1985^4^,^71^ and 1993^1^,^55^ (Table 2). The SF-36^56^ validation also has been studied, but its validity as a extremity FO measure in this population is questionable.^1^ In addition, the validity of the Short Musculoskeletal Function Assessment (SMFA) questionnaire,^57^,^58^ the Foot Function Index (FFI),^59^,^60^ the Karnofsky score,^61^ and the Reintegration to Normal Living Index (RNL)^62^,^63^ measures also has been studied. However, these measures were studies in a different population of patients or as a general FO measure, or both. No studies on the validity or reliability of the modified International Society Of Limb Salvage (ISOLS)/MSTS 1993^27^ scoring system or the modified Convery scale^64^ were found.

### Functional Outcome

The TESS, MSTS 1993, or MSTS 1987 results were presented in 23 of the 37 studies. In some studies, not all the required results (mean or SD of FO results, number of lower- vs upper-extremity patients, number of STS vs other tumors) were presented. Additional information was requested from 15 corresponding authors. Five replied, with two supplying the requested additional information.

Of the 23 studies, 3 were excluded from our FO report. The reasons were as follows: only the difference in means was reported;^18^ several TESS scores from the same patients were included (in lower-extremity patients, a mean of 2.6 TESS results for one patient was included);^21^ and two studies used the same data set (a study by O’Sullivan was excluded).^39^,^52^ Means and SDs for a study by Saebye et al.^19^ were calculated using median and IQR results.^65^,^66^ The SDs for studies by Friedmann et al.^28^ and Pradhan et al.^33^ were approximated based on range using the range rule calculation formula (SD = [max − min]/4). The pre- and postoperative function results are presented in Table 3.

**Table 3:** Pre- and postoperative function scores. Results of the TESS, MSTS 1993 and MSTS 1987 measures

Author	Year	LE patients (*n*)	Mean TESS (SD)^a^	Mean MSTS 1993 (SD)^a^	Mean MSTS 1987 (SD)^b^	Comments
Preoperative						
Rivard	2015	35	79.8 (20.6)	78.8 (20.7)	NA	FO results including UE STS patients (*n* = 15). Results available for MSTS from 45 and for TESS from 48 patients
Gerrand	2004	207	83.1 (20.1)	87.9 (19.6)	NA	Results available for MSTS from 203 and for TESS from 172 patients
Davis	2002	147	84.4 (19.0)	NA	26.8 (4.4)	FO results including UE STS patients (*n* = 38)
Postoperative						
Saebye	2017	90	94 (11.3)	92.8 (13.6)	NA	FO results including UE STS patients (*n* = 38)
Harati	2016	120	63.8 (17.0)	NA	NA	FO results available for 30 patients
Chang	2016	129	NA	80.2 (NP)	NA	FO results including UE STS and other tumors. Excluded from the pooled mean and SD analysis due to missing SD data
Furtado	2015	37	56.4 (23.3)	NA	NA	TESS results including bone sarcoma patients (*n* = 63)
Rivard	2015	35	87.1 (16.6)	88.8 (11.9)	NA	FO results including UE STS patients (*n* = 15). Results available for MSTS from 37 and for TESS from 41 patients
Kang	2014	104	NA	85.4 (13.9)	NA	FO result including UE STS patients (*n* = 44)
Townley	2013	21	84.76 (NP)	NA	29.73 (NP)	FO results including bone sarcoma patients. Excluded from the pooled mean and SD analysis due to missing SD data
Friedmann	2011	204	89.4 (32.4–100)^c^	NA	32 (11–35)^c^	FO results including UE STS patients (*n* = 59)
Thacker	2008	52	NA	83.3 (11.5)	NA	FO results available for 30 sarcoma patients
Tsukushi	2008	25	NA	70 (NP)	NA	
Hoy	2006	70	NA	90.3 (NP)	NA	FO results including UE STS (*n *= 6) and bone sarcoma (*n* = 39) patients
Pradhan	2006	184	77 (23–100)^c^	NA	NA	FO results available for 70 patients
Nelson	2006	48	85.1 (19.3)	NA	NA	FO results available for 34 patients
Davis	2005	100	83.2 (21.8)	NA	28.9 (9.2)	FO results including UE STS patients (*n* = 29)
Gerrand	2004	207	82.7 (17.7)	85.8 (19.0)	NA	Results available for MSTS from 189 and for TESS from 155 patients
Rachbauer	2003	29	NA	88.5 (NA)	NA	FO results including UE STS (*n* = 6) and other anatomic location sarcomas (*n* = 4)
Davis	2002	147	80.3 (21.1)	NA	28.6 (7.8)	FO results including UE STS patients (*n* = 38). Results available for MSTS from 163 and for TESS from 156 patients
Davis	2000	172	82.7 (18.7)	84.8 (20.4)	30.0 (6.2)	
Davis	1999	29	81.6 (17.8)	NA	NA	FO results including bone sarcoma patients (*n* = 7)
Colterjohn	1997	29	NA	NA	31.4 (4.6)	FO results available for 26 patients

*TESS* Toronto extremity salvage score, *MSTS* Musculoskeletal Tumor Society, *LE* lower-extremity, *SD* standard deviation, *NA* not available, *NP* not presented, *UE* upper extremity, *STS* soft tissue sarcoma, *FO* functional outcome

^a^Scores are presented as range of 0 (minimum) to 100 (maximum)

^b^Scores are presented as range of 0 (minimum) to 35 (maximum)

^c^Range (min–max)

After study selection and additional gathering of information, some studies still included upper-extremity or bone sarcoma patients.

**Fig. 2:**
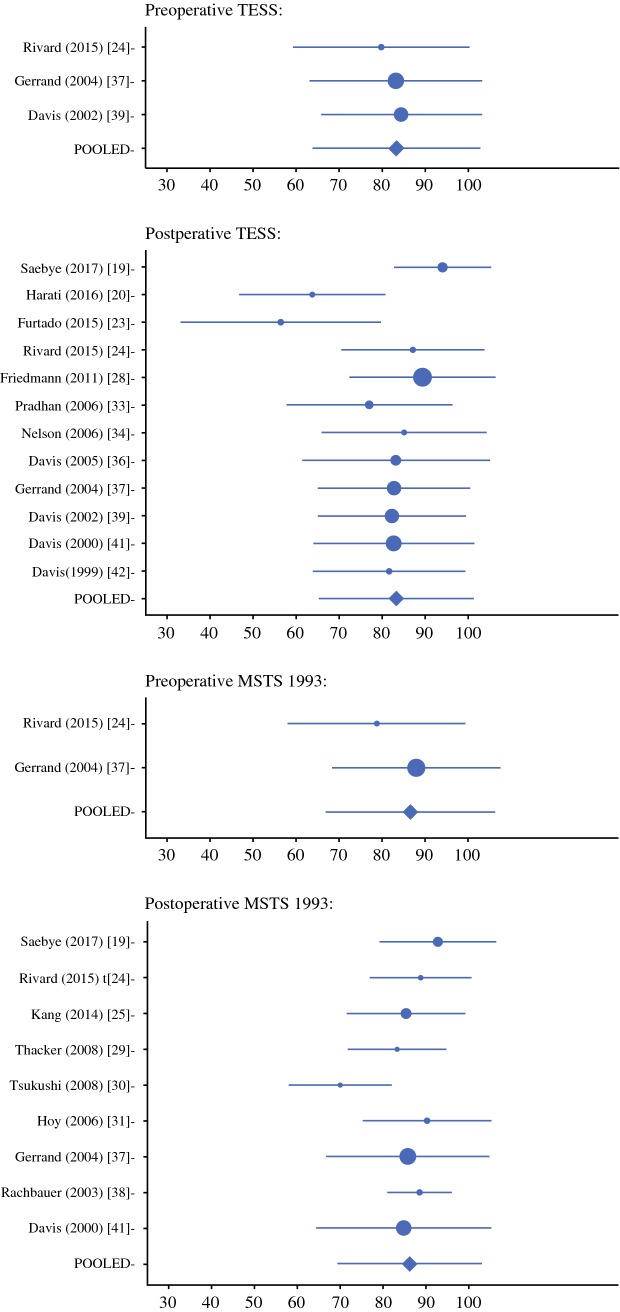
Pre- and postoperative TESS and MSTS 1993 pooled mean and SD results

The pooled mean and SD were calculated for TESS and MSTS 1993 results. Of 19 studies, 2 were excluded due to missing SD data. The pre- and postoperative TESS and MSTS 1993 pooled mean and SD results are presented in Fig. 2. Because not all the publications included only lower-extremity patients, TESS results for both lower- and upper-extremity STS patients were included in 2 of 3 preoperative and 5 of 12 postoperative studies. Similarly, MSTS 1993 results included both lower- and upper-extremity STS patients in 1 of 2 preoperative and 5 of 9 postoperative studies. In 3 of 17 studies, the TESS (*n* = 2) and MSTS 1993 (*n* = 1) results included other extremity tumor patients in addition to STS patients. The number of included patients is described in Table 3.

The preoperative pooled mean TESS and MSTS 1993 scores were respectively 83.3 and 86.6, and the postoperative scores were respectively 83.3 and 86.2 (out of 100). In the pooled mean and SD analysis, the proportion of lower-extremity and STS patients were respectively 88% and 94%.

As a sensitivity analysis, the mean overall postoperative FO in publications including also upper-extremity or bone sarcoma patients was investigated. In publications including upper-extremity patients (7 of 17 publications), the mean overall TESS score was 86.7 (5 publications), and the MSTS score was 89.0 (5 publications). In publications that included also bone sarcomas (3 of 17 publications), the mean overall TESS score was 63.1 (2 publications), and the MSTS score was 90.3 (1 publication).

### Quality of Publications

The mean MINORS score was 62.2 (median, 62.4; range, 31–92). For the three RCTs, the respective scores were 96,^39^ 96,^52^ and 88.^36^ For the observational studies, the mean MINOR score was 60.2 (median, 62.5; range, 31–92). The MINORS scores for each study are presented in Table 1.

## Discussion

Based on the current systematic literature review, the most frequently used measurement types used to measure FO for surgically treated adult lower-extremity STS patients are clinician-reported outcome and PRO measurements. The most frequently used measures are the TESS and the MSTS 1993 questionnaires. Of the 10 previously reported measures, 3 were proven to provide valid scores for lower-extremity sarcoma patients. Most of the studies on lower-extremity STS FO have poor methodologic quality.

### Methods and Measures of FO

The majority of the included studies used clinician-reported outcome measurement. In the last 10 years, the use of PRO measurements has increased. Furtado et al.^2^ considered performance outcome an important component of FO in sarcoma patients. This clearly is a minority position in their study because no performance outcome measurement type was found. Performance outcome measurement type has been used more frequently in publications with smaller study samples.^2^ This may be due to the greater time requirements and equipment and personnel costs required for performance outcome measurements.^2^

In the literature review of Furtado et al.^2^ that investigated performance outcome measurement techniques, the study size ranged from 4 to 82 patients. In the current review, one of the inclusion criteria was a minimum of 20 lower-extremity STS patients. In our study, the sample size ranged from 25 to 728 patients.

When FO is assessed in a study, it is important to avoid loss to follow-up evaluation. Loss to follow-up evaluation less than 5% is indicated in the MINORS publications’ quality measurement tool as an indicator of a good-quality study.^14^ More important than the measurement type is that the outcome measure provide valid and reliable scores in measuring FO. However, the type of measurement should be chosen based on the objectives of the assessment, the available resources, and the aspects of FO that are of interest.

According to the current review, the TESS and MSTS 1993 questionnaires have been used most frequently. Tang et al.^3^ observed that the most frequently used outcome measures were the TESS, the MSTS 1987, and the SF-36. However, they observed that the TESS was used only four times, the MSTS 1987 three times, and the MSTS 1993 two times. They also found that when the MSTS was used, the 1987 version was preferred. Likewise, Groundland et al.^7^ found that the MSTS is the most widely used FO measure for pediatric patients after limb-preservation surgery.

Winnette et al.^9^ investigated patient experience with STS in all anatomic locations, including abdominal sarcomas. They found that in extremity patients, the RNL and the TESS were used three times each. The MSTS questionnaire was not presented in their review because it examined only PRO measures.

Wilson et al.^8^ found that the MSTS was the most frequently used measure and that the TESS was presented only once for pelvic sarcoma patients. Some studies have used both the older and newer versions of the MSTS questionnaires in the same study.^18^,^41^ Other measures such as the SF-36, the FFI, the Karnofsky score, the RNL, the SMFA, the modified MSTS 93/ISOLS, and the modified Convery scale^64^ have been used but much less frequently.

The MSTS 1987^54^ is a clinician-reported outcome assessment that evaluates seven parameters of FO (mobility, pain, stability, deformity, strength, functional, and emotional acceptance). The MSTS 1993,^55^ a revised version of the MSTS 1987, also is assessed by physicians. However, the MSTS 1993 is more limb-specific than the older version and includes six parameters. Pain, function, and emotional acceptance are measured for both extremities. For the lower-extremity, use of walking aids, gait, and walking are evaluated. Hand positioning, dexterity, and lifting ability are evaluated for the upper extremity.

The TESS was developed for limb sarcoma patients. As a PRO questionnaire, it measures physical disability and performance in activities of daily living.^53^ Janssen et al.^67^ found that the TESS has adequate coverage and is more reliable than the MSTS questionnaire. In addition, they found that the MSTS score is the least reliable, as indicated by a high standard error of measurement for the complete range of ability scores. Although both versions of MSTS are the most frequently used tool for assessing FO, the TESS tool is more frequently used than the two MSTS versions separately. The TESS also was developed later than the MSTS measures.^53^–^55^

The study by Tang et al.^3^ included only articles published in last 10 years. The TESS was used in four publications, the MSTS 87 in three publications, and the MSTS 93 in two publications. Because of the relatively wide use of MSTS scores, the MSTS questionnaires permit comparison of results more easily and widely than in other studies. On the other hand, the MSTS questionnaire must be completed by a clinician, thus limiting its use in studies with larger samples.

The TESS questionnaire was designed to be completed by patients and can therefore be administered by mail or electronically. This is important particularly in long-term follow-up studies. Using the TESS may avoid the need for a physician consultation, thus saving resources. Using PRO measures may make it easier for patients to participate in the study. Also, minimal clinically important differences (MCIDs) are calculated for the TESS.^68^ Because the MSTS measure was developed by orthopedics for surgically treated bone and soft tissue musculoskeletal tumors, it may not capture the effects of radiotherapy, chemotherapy, and other factors that also affect FO.^16^,^36^,^48^,^49^,^69^ Although all FO measurements have limitations, the use of standardized instruments is important. Using the TESS and MSTS measures allows for benchmarking and comparison of results with other studies.

According to the results of our literature review, most studies used only one FO measure. The use of more than one FO measure provides more precise information on FO. For example, the TESS measures activity limitations, whereas the MSTS measures impairment in extremity sarcoma patients.^53^,^55^ On the other hand, using too many time-consuming questionnaires could lead to decreased participation and loss to follow-up evaluation. In addition, using more than one FO measuring tool might not be clinically relevant.^70^ Study participants should be informed about the patient burden, including how many items must be completed, how long it takes to complete the questionnaires for participation in the assessment, and how many assessments are made. Questionnaires implemented in a clinical study should be carefully chosen to ensure that they are fit for the purpose.^71^–^74^

Most retrospective studies required a minimum follow-up period of 1 year after surgery. In addition, FO showed a decrease up to 6 months after the surgery. Beyond 1 year, FO plateaued before the scores returned to approximate pre-treatment levels 1 year after surgery.^39^,^43^,^75^

Rivard et al.^24^ did not observe significant changes in the TESS and MSTS scores from the preoperative period to 6 months after surgery. By 12 months, the scores showed significant improvement. This is an important factor when prospective data measurement in retrospective samples is planned. In cross-sectional studies and other prospective studies, it is important to consider measuring FO at least 1 year after the surgery, in addition to other measurement time points.

### Validity of Measures

The validity of a measurement tool is a multi-dimensional term. The most important measurement property is content validity. The measure should be relevant, comprehensive, and comprehensible with respect to the construct of interest and the study population.^76^ Structural validity is the degree to which the scores adequately reflect the dimensionality of the construct to be measured.^39^,^77^ This review considered the measure to be validated when it was tested in lower-extremity patients and reported to be a valid measurement tool.

Half of all the studies reviewed (23/37 studies) used validated tools. In 1999, Davis^4^ found that studies often did not use standardized, validated measures. Several measurement tools currently in routine use had been available for only a few years in 1999. Based on our review and on the previous literature, it seems that although the psychometric properties of several PRO and clinician-reported outcome measures have been extensively studied in the last decade, performance outcome measures lack quality in this field.^2^

In a systematic review of objective measurement methods, Furtado et al.^2^ found that only a few studies investigated aspects of validity of outcome measures. For example, they found that only 1 in 18 studies investigated reliability. They concluded that this raises questions about the accuracy of the objective (in our terminology, performance outcome) measures and veracity of the results.^2^

### FO of Lower-Extremity STS Patients

The data sample in the current review included a heterogeneous group of lower-extremity STS patients. According to the current review and analysis, the postoperative FO for patients is relatively good. The mean postoperative FO measured by MSTS for patients with extremity osteosarcoma is reported to range between 40% and 76.6%.^78^ In pediatric bone sarcoma patients, the reported postoperative MSTS mean scores ranged from 76% to 82.5%.^7^ In a review of pelvic sarcoma patients, the mean MSTS score was 65%.^8^

Amputation seems to decrease FO.^23^,^78^ Our FO data included relatively few articles on amputation. The MSTS 1993 FO analysis did not include any articles and the TESS analysis included only two articles on amputations.^23^,^42^ In the work of Davis et al.^42^ the mean TESS score for the patients with amputations was 74.5 versus 85.1 for the patients with limb-sparing procedures. In the study by Furtado et al.^23^ the mean TESS score was only 56.4. Han et al.^78^ performed a meta-analysis of osteosarcoma patients and observed that the number of amputation patients was relatively higher. The mean MSTS for the amputation patients ranged from 41.1% to 71% versus 70% to 76.6% for the patients with limb-sparing procedures, which is lower than the result of the current review. As expected, some studies show that amputation decreases FO,^23^,^78^ whereas others have failed to show a significant difference compared with limb-sparing treatment.^79^–^81^ Based on this review, lower-extremity STS patients seem to achieve preoperative function levels postoperatively during long-term follow-up evaluation (> 1 year).

### The Quality of the Publications Reporting FO

This review included different types of studies examining varied methodologic quality. The use of the MINORS tool showed that most studies investigating lower-extremity STS FO are lacking in methodologic quality (median MINORS score, 62.4%; range, 31–92).

This is the first systematic review of FO measurement to focus on lower-extremity STS patients. The strengths of this review were the use of PRISMA guidelines and the methodologic quality assessment for this topic. The main weakness of the current review was the nonstructural search strategy for validity of FO measurement tools used. In addition, one exclusion criterion was to have “fewer than 20 lower-extremity STS patients in the study (considered a pilot study).” This might have excluded some relevant studies with small samples. Because the authors attempted to overview the existing literature on FO measurement of lower-extremity STS patients as thoroughly as possible, they included some studies containing small amounts of upper-extremity and bone sarcomas. Because the number of non–lower extremity STS patients in the reviewed studies was small (88% lower-extremity and 94% STS patients), and because the sensitivity analysis presented similar FO results for publications reporting on upper-extremity or bone sarcoma patients, the effect on FO results was small.

## Conclusion

The most frequently used FO measurements for surgically treated adult lower-extremity STS patients are clinician-reported outcome and PRO measurements. The most widely used measure is the patient-reported TESS instrument, which has been shown to produce reliable and valid scores in assessing FO for lower-extremity sarcoma patients. Using the TESS and MSTS measures allows for benchmarking and comparison of results with other studies. Functional outcome scores seem to return to pretreatment levels 1 year after surgery. Thus, measurement of FO also should be performed at least once 1 year after surgery or later in addition to other time points. This review indicates that quality is lacking in FO studies examining surgical treatment of lower-extremity STS.
